# Supplementary material for: Initial Steps for Quality Improvement of Obesity Care Across Divisions at a Tertiary Care Pediatric Hospital
Source: Int J Environ Res Public Health. 2014 Sep 17;11(9):9680–93. doi: 10.3390/ijerph110909680 (PMC4199043; doi:10.3390/ijerph110909680)
Supplement: Supplementary File 1 [file ijerph-11-09680-s001.pdf]

*Supplementary Information*

# Initial Steps for Quality Improvement of Obesity Care Across Divisions at a Tertiary Care Pediatric Hospital

---

**Characterizing Practice Systems**
**Structured Interviews with Key Informants of Respective Clinics [1–3]**

Introductory Script

"Thank you for your participation. The following questions aim to understand the processes of care that subspecialists use to identify overweight/obesity and elevated blood pressure across their practices."

**PRACTICE______________________________________________________________**

**Resources**

1. Do clinics in your division provide educational materials (ie handouts/brochures) about obesity management to families of obese patients?

    Yes, across the division

    Yes, certain clinics:____________________________________________

        Widely Available Materials (*i.e.* CDC, *etc.*)

        Division-Specific Materials (*i.e.* Epic patient education materials)

        Other______________________________________________

    No

    Don't know

2. Are there available resources within clinics in your division to help you identify and manage obese patients?

    Yes, across the division

    Yes, certain clinics:____________________________________________

        EPIC Smart Texts

        Referral to special clinics for obese patients (ie fatty liver clinic)

        Other______________________________________________

No

Don’t know

3. Does your division have an assigned dietitian who sees pediatric patients in the outpatient setting?

Yes

If yes, how many ½ days per week __________

No

Don’t know

4. Is there a dietitian available in clinics within your division for counseling of obese patients identified in your practice?

Yes, specify clinics:_____________________________________________

No

Don’t know

**Measurements**

5. At which types of visits are patients’ height or length routinely measured at clinics within your division?

Initial Consultation    Follow-up Consultation

Initial Well-Child    Follow-up Well-Child

Specifics:____________________________________________________________

5b. Who takes these measurements?

Doctor    Nurse    Nursing assistant/auxiliary    Other

5c. Who interprets these measurements?

(Clarification: Interpret refers to reading from record and assessing percentile)

Doctor    Nurse    Nursing assistant/auxiliary    Other

6. At which types of visits are patients’ weight routinely measured at clinics within your division?

Initial Consultation    Follow-up Consultation

Initial Well-Child    Follow-up Well-Child

Specifics:____________________________________________________________

6b. What are patients usually wearing when they are weighed?

Hospital Gown

Regular Clothes

Other, specify ______________________________________________

6c. Who takes these measurements?

Doctor    Nurse    Nursing assistant/auxiliary    Other

6d. Who interprets these measurements?
(Clarification: Interpret refers to reading from record and assessing percentile)

Doctor    Nurse    Nursing assistant/auxiliary    Other

7. At which types of visits are patients' blood pressure routinely measured at clinics within your division?

Initial Consultation    Follow-up Consultation

Initial Well-Child    Follow-up Well-Child

Specifics:____________________________________________________

7b. Who takes these measurements?

Doctor    Nurse    Nursing assistant/auxiliary    Other

7c. Who interprets these measurements?
(Clarification: Interpret refers to reading from record and assessing percentile)

Doctor    Nurse    Nursing assistant/auxiliary    Other

**Methods**

8. Is there anything special about how clinics in your division obtain height or length measurements?

Yes, describe:______________________________________________

Written protocol
If written protocol, please provide copy of the protocol.

No

Don't know

9. Is there anything special about how clinics in your division obtain weight measurements?

Yes, describe:______________________________________________

Written protocol
If written protocol, please provide copy of the protocol.

No

Don't know

10. Is there anything special about how clinics in your division obtain blood pressure measurements?

Yes, describe:______________________________________________

Written protocol

If written protocol, please provide copy of the protocol.

No

Don't know

**Data Entry**

11. Who is responsible for routinely entering height/weight measurements into Epic?

Clinician

Nurse

Nursing assistant/auxiliary

Other (please specify):____________________________________

Don't know

12. Who is responsible for routinely entering blood pressure measurements into Epic?

Clinician

Nurse

Nursing assistant/auxiliary

Other (please specify):____________________________________

Don't know

**PROTOCOLS**

13. Are there any strategies/protocols in place within clinics in your division **<u>to identify</u>** overweight/obese patients?

Written Protocol

Describe:________________________________________

Informal Policies

Describe:________________________________________

No

14. Are there any strategies/protocols in place within clinics in your division **<u>to manage</u>** overweight/obese patients?

Written Protocol

Describe:________________________________________

Informal Policies

Describe:________________________________________

No

15. Are there any strategies/protocols in place within clinics in your division **to identify** patients with elevated blood pressure?

    Written Protocol

    Describe:___________________________________________

    Informal Policies

    Describe:___________________________________________

    No

16. Are there any strategies/protocols in place within clinics in your division **to manage** patients with elevated blood pressure?

    Written Protocol

    Describe:___________________________________________

    Informal Policies

    Describe:___________________________________________

    No

17. Do clinics in your division screen for obesity related comorbidities?

    Yes

    If yes, what is routinely screened for and within which clinics? ____________

    No

    Don't Know

If yes, answer Questions 18 and 19.

18. Please describe what is usually done by providers in clinics within your division when a patient is identified with an obesity related comorbidity. (open ended)

    Follow-up Management:

    Return to PCP

    Referral Out, specify:____

    Other:

19. Are there protocols in place for the management of obesity related comorbidities?

    Yes

    Written Protocol

    Described:___________________________________________

    Provided

    Informal Policies

    Other: ___________________

No

Don't know

**PHILOSOPHY**

**Roles**

20. What do you think is the role of clinics within your division in **identifying** overweight or obese patients?

    Open ended, to be categorized

    ________

    ________

    No role

21. What do you think is the role of clinics within your division in **managing** overweight or obese patients?

    Open ended, to be categorized

    ________

    ________

    No role

22. What do you think is the role of clinics within your division in **identifying** patients with obesity-related comorbidities?

    Open ended, to be categorized

    ________

    ________

    No role

23. What do you think is the role of clinics within your division in **managing** patients with obesity-related comorbidities?

    Open ended, to be categorized

    ________

    ________

    No role

24. What do you think is the role of clinics within your division in **identifying** patients with elevated blood pressure?

    Open ended, to be categorized

    ________

    ________

    No role

25. What do you think is the role of clinics within your division in **managing** patients with elevated blood pressure?

    Open ended, to be categorized

    ________

    ________

    No role

**Barriers**

26. What barriers do you perceive to identifying obese/overweight patients within clinics in your division? (Open ended, to be categorized)

    ________

    ________

    No barriers

    26b. Do you have any ideas on how to address these barriers?

27. What barriers do you perceive to providing care to obese/overweight patients within clinics in your division? (Open ended, to be categorized)

    ________

    ________

    No barriers

    27b. Do you have any ideas on how to address these barriers?

28. What barriers do you perceive to identifying patients with elevated blood pressure within clinics in your division? (Open ended, to be categorized)?

    ________

    ________

    No barriers

    28b. Do you have any ideas on how to address these barriers?

29. What barriers do you perceive to providing care for patients with elevated blood pressure within clinics in your division? (Open ended, to be categorized)

________

________

No barriers

29b. Do you have any ideas on how to address these barriers?

**INTERESTS**

**30.** What resources or strategies do you think would help clinics in your division identify and address patients who are overweight or obese? (asked as an open ended question)

**Y N**

Handouts/Brochures

Changes in EPIC Medical Record Forms

Meetings/Educational Courses

Specific Protocols/Guidelines for Identification and Treatment

Specific Protocols/Guidelines for Referral

Expanded Referral Options for Obesity Care within Lurie Children's

Other, please specify:_____________________________________________

**31.** What resources or strategies do you think would help clinics in your division identify and address patients with elevated blood pressure? (asked as an open ended question)

**Y N**

Handouts/Brochures

Changes in EPIC Medical Record Forms

Meetings/Educational Courses

Specific Protocols/Guidelines for Identification and Treatment

Specific Protocols/Guidelines for Referral

Expanded Referral Options for Blood Pressure Management within Lurie Children's

Other, please specify:_____________________________________________

**Survey Adapted From:**

1. Ariza, A.J.; Laslo, K.M.; Thomson, J.S.; Seshadri, R.; Binns, H.J.; Group, P.P.R. Promoting Growth Interpretation and Lifestyle Counseling in Primary Care. *J. Pediatr.* 2008;10.

2. Barlow, S.E.; Dietz, W.H.; Klish, W.J.; Trowbridge, F.L. Medical Evaluation of Overweight Children and Adolescents: Reports From Pediatricians, Pediatric Nurse Practitioners, and Registered Dietitians. *Pediatrics* **2002**, *110*, 222–228.
3. Leslie, W.S.; Hankey, C.R.; McCombie, L.; Lean, M.E. Weight management: A survey of current practice in secondary care NHS settings in 2004. *J. Eval. Clin. Prac.* **2005**, *11*, 462–467.

© 2014 by the authors; licensee MDPI, Basel, Switzerland. This article is an open access article distributed under the terms and conditions of the Creative Commons Attribution license (http://creativecommons.org/licenses/by/3.0/).
